# Sugar Esters of Fatty Acids: Chemo-Enzymatic Synthesis and Biological Activity

**DOI:** 10.3390/molecules30153123

**Published:** 2025-07-25

**Authors:** Kinga Hyla, Tomasz Janek

**Affiliations:** Department of Biotechnology and Food Microbiology, Wrocław University of Environmental and Life Sciences, 51-630 Wrocław, Poland; kinga.hyla@upwr.edu.pl

**Keywords:** synthesis, surfactant, biosurfactant, green chemistry, sugar ester, solvent system, DES, biological activity

## Abstract

Human applications of surfactants have been diverse, from their initial use as detergents to their subsequent utilization in a multitude of other fields, including medicine, lubricants, cosmetics, and even assisted oil recovery. Nevertheless, the most significant challenge lies in the synthesis of surfactants. A particular challenge is the purification of compounds following chemical synthesis, as well as the toxic effect of the solvents used. Consequently, there is a growing need for more environmentally friendly solutions, namely solvents that are less toxic and more biocompatible, as well as reactions in which an enzyme serves as a catalyst. This review examines the various methods of synthesizing sugar esters and glycolipids, evaluating their respective advantages and disadvantages.

## 1. Introduction

Surfactants are a versatile group of organic compounds with a wide range of applications across various industries. They are indispensable in a plethora of products, including laundry detergents, emulsifiers, foaming agents, and wetting agents. Additionally, they are utilized in various industries, such as paints, oil recovery, printing, cosmetics, pharmaceuticals, and food [1]. These compounds can be synthesized through two distinct methods: the first involves the use of petrochemical or oleochemical precursors, while the second employs the biological synthesis of microorganisms, including bacteria, fungi, and yeasts. This process results in the production of biosurfactants or bioemulsifiers [2,3].

Synthetic surfactants are the dominant choice in industrial applications due to their efficacy and extensive availability [4]. However, the extensive use of these substances presents a significant environmental risk, particularly to aquatic ecosystems [5,6,7]. Over the past two decades, global surfactant production has increased significantly, from 7 million tons annually 20 years ago [5], increasing to 12.5 million tons in 2006 [8] and to 14.1 million in 2017 [1]. This production is projected to grow by 18% by 2022, according to the latest estimates [9]. The global market value of surfactants was estimated at USD 39.9 billion in 2019 and is projected to reach USD 52.4 billion by 2025, with the impact of the COVID-19 pandemic driving further demand [1]. The synthesis of surfactants typically involves chemical reactions that combine a hydrophobic chain with a hydrophilic group. Synthetic surfactants are classified based on their ionic charge as cationic, anionic, nonionic, and zwitterionic or amphoteric surfactants [3]. The most common anionic surfactants consist of sulfate, sulfonate, phosphate, and carboxylate salts. In this category, organ sulfates such as sodium lauryl sulfate (SLS) can be found. SLS is a mixture of sodium alkyl sulfates, with sodium dodecyl sulfate (SDS) being the main component [10,11]. Additionally, SDS and related potassium and ammonium salts are also present. Cationic surfactants, including protonated primary, secondary, and tertiary amines, as well as quaternary ammonium salts, are commonly used [12]. Among these, cetrimonium bromide (CTAB) is the most frequently used. The following chemicals have been used: cetylpyridinium chloride (CPC), benzalkonium chloride (BAC), benzethonium chloride (BZT), dimethyldioctadecylammonium chloride (DODMAC), and dioctadecyl dimethylammonium bromide (DODAB) [3,13]. An interesting group of biosurfactants are amphoteric surfactants. These compounds are characterized by the presence of a hydrophobic hydrocarbon chain and hydrophilic centers that exhibit both positive and negative charges. These centers are connected by a spacer group, which facilitates their interaction with water molecules. Consequently, this class of surfactant preserves overall charge neutrality. The properties of these compounds are determined primarily by the length of the hydrophobic hydrocarbon chain, the number of methylene segments in the spacer group, the presence of positive and negative charges, and their relative positions [14]. The following substances are included: diethylamino-lauryl itaconate (DEALT) through the synthesis of lauryl itaconate (LI)-based anionic polymerizable surfactant; amphoteric polymer auxiliary with methyl acrylic acid; and sodium methylallyl sulfonate. The following substances were examined: dimethyl diallyl ammonium chloride (DMDAAC) [14], poly(tetrahydrofuran glycol) (PTMG), 2,4-dihydroxybenzaldehyde (DDBA), 2,2-dihydroxymethyl propionic acid (DMPA), and N-methyl diethanolamine (N-MDEA).

Carboxylate-based surfactants are primarily employed in the formulation of soaps, while sulfonate-based surfactants, which exhibit solubility in both acidic and alkaline media, find application as detergents, dispersants and flocculants in suspensions [15]. Conversely, sulfonates exhibit superior chemical stability relative to sulfates, consequently demonstrating enhanced resistance to hydrolysis under both low and high pH conditions. Phosphate ester surfactants are extensively utilized in the textile manufacturing industry for the purpose of cleansing oil and wax, due to their exceptional emulsifying properties, which remain effective even within strongly alkaline contexts. However, their resistance to hardness, characterized by elevated calcium and magnesium concentrations, renders them unsuitable for certain applications, such as oil extraction [16]. Despite the prevalence of anionic and nonionic surfactants, cationic biosurfactants have a niche application in which their ability to modify the surface properties of solids is exploited [17]. Furthermore, their utilization extends to the domains of corrosion inhibitors, fuel and lubricant additives, and bactericides [18]. Amphoteric surfactants are generally employed in conjunction with other surfactants (as cosurfactants) to modify and enhance the performance of the primary surfactant. This is due to their capacity to bind to both positively and negatively charged molecules and their propensity to alter micelle structure. These materials have a wide range of applications in the fields of cleaning and emulsions [19]. Moreover, in recent years, the popularity of sugar-based surfactants with properties similar to those of the previously mentioned surfactants has been increasing [20].

Carbohydrates, constituting 95% of the world’s biomass, have become a focal point for producing sugar-based surfactants. These surfactants are increasingly popular due to their high production yields and lower toxicity compared to traditional surfactants [10,21]. Sorbitan esters, sucrose esters, alkyl polyglycosides (APG), and fatty acid glucamides are examples of sugar-based surfactants that are gaining popularity due to their improved performance, consumer health benefits, and environmental friendliness compared to some traditional formulations. Sorbitan esters, commercially known as Spans, are highly hydrophobic and tend to form water-in-oil emulsions [22]. The global sorbitan ester market is forecast to reach USD 756.4 million in 2021. Sales of sorbitan esters are expected to grow by 5.6% compound annual growth rate (CAGR) between 2021 and 2031 [21]. Sucrose esters have varying degrees of hydrophilicity depending on the level of esterification in the compound. Sugar-based surfactants are now commercially available. These include sucrose esters (Sisterna^®^, Ryoto^®^, Sucrosoft^®^), sorbitan esters (Span^®^), rhamnolipids, and sophorolipids (Bio Base Europe and R-95, SL-P (Sopholiance^®^). Due to their diverse scale of hydrophobicity, esters are used in a wide range of applications, from personal care to emulsifiers to specialty detergents [21,23]. Sucrose esters, which vary in hydrophilicity based on esterification, are used in a wide range of applications, including personal care products and specialty detergents. Their market value was USD 72 million in 2022, with projections reaching USD 111.6 million by 2030 [24].

**Figure 1:**
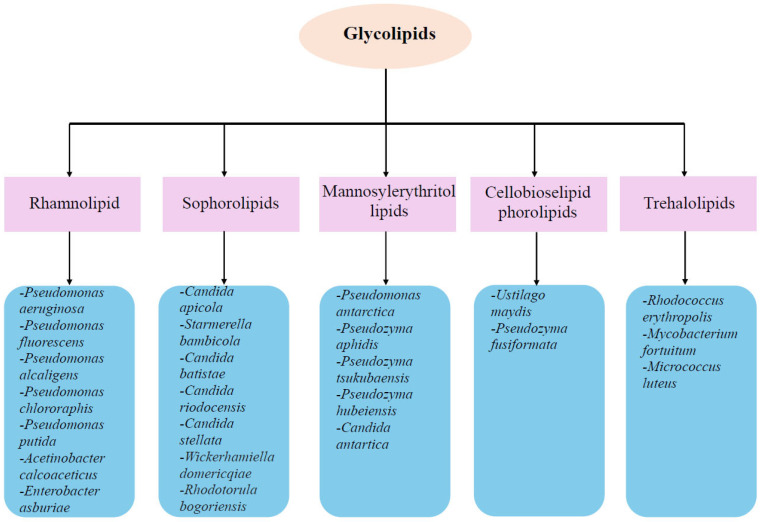
Main classes of microbial sugar-based biosurfactants and their representative producers (blue boxes). Each group corresponds to a distinct glycolipid type with unique structural and functional properties.

**Figure 2:**
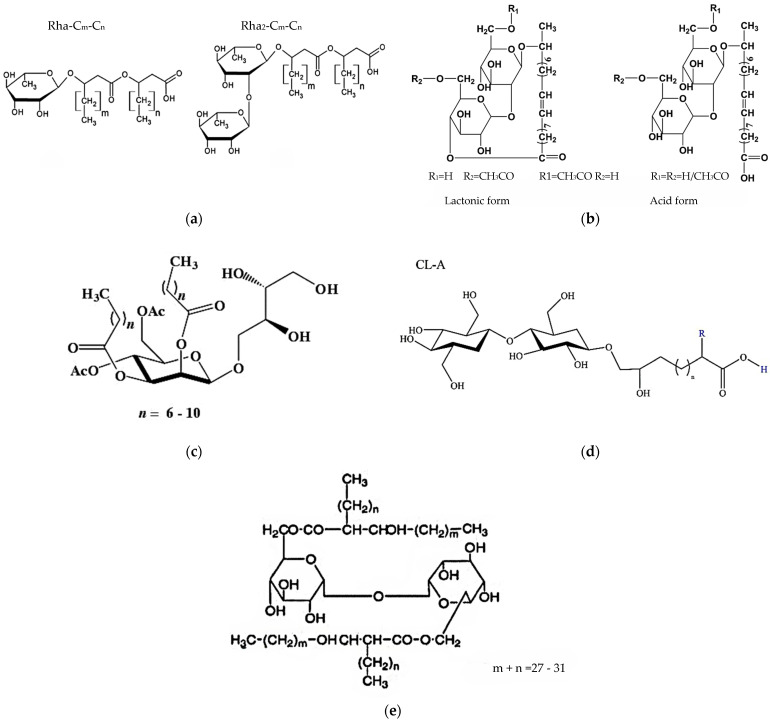
The general structure of glycolipids was drawn using ChemSketch software version ACD/Labs 6.00). (**a**) Rhamnolipids [37], (**b**) sophorolipids [38], (**c**) mannosylerythritol lipids [39], (**d**) cellobiose lipids, (**e**) trehalolipids [40].

Microbial biosurfactants are secondary metabolites produced by a variety of microorganisms, including bacteria, fungi, and yeast (Figure 1). They are typically produced extracellularly, although some microorganisms can produce biosurfactants that are bound to the plasma membrane [3,25,26,27,28]. Biosurfactant-producing microorganisms can produce surface-active compounds with high or low molecular weights. High-molecular-weight biosurfactants are more effective at stabilizing oil–water emulsions, while low-molecular-weight biosurfactants are better at reducing surface tension [29,30]. The hydrophilic molecule of biosurfactants is composed of mono-, di-, or polysaccharides, anions, or cations. Meanwhile, the hydrophobic part of the molecule comprises both saturated and unsaturated fatty acids [31]. Microbial surfactants are more biodegradable, less toxic, and have better stability and foaming properties under different environmental conditions than chemically synthesized surfactants [32,33,34]. One of the most widely used groups of biosurfactants in the industry is glycolipids. They are lipid derivatives and consist of long chains of sugars containing hydroxy aliphatic or aliphatic acids. Glycolipids represent a group of biosurfactants in which the head consists of a polar sugar group. This diverse group of surfactants includes rhamnolipids, sophorolipids and their derivatives, mannosylerythritol lipids, cellobiose lipids, and trehalolipids (Figure 2) [29,35,36].

The chemo-enzymatic synthesis of sugar-based surfactants has increased significantly in recent years due to their advantageous properties, including high biodegradability, low toxicity, and high environmental compatibility. Moreover, this new class is more advantageous considering that they are derived from natural and renewable raw materials. The hydrophilic part of a sugar-based surfactant contains a sugar molecule, and the hydrophobic part is usually a fatty acid. Several classes of sugar-based non-ionic surfactants are already available on the market, including APG, sucrose esters, and sorbitan esters. The synthesis of these surfactants by chemical and enzymatic means has already been described in the literature. The chemical route is non-selective, producing several by-products [20], whereas the enzymatic route is more specific and allows the production of monosubstituted sucrose esters or ethers [20,41,42,43]. This review examines the synthesis and properties of sugar-based surfactants, focusing on their potential antimicrobial, antibacterial, antifungal, and anticancer activities. These properties highlight the growing significance of these compounds in a variety of industrial applications, as well as their potential to replace conventional surfactants, thereby contributing to more sustainable and environmentally friendly practices.

## 2. Synthesis

The structures of sugar-based surfactants are diverse, encompassing glycoamides, glycoesters, glycosides, and other derivatives. This diversity of structure is reflected in the diverse properties of these surfactants [44]. The esterification process has traditionally been carried out using chemical catalysts. Nevertheless, it is becoming increasingly evident that the utilization of chemical catalysts is associated with several disadvantages. These include environmental pollution, the necessity of adhering to strict process parameters, and the difficulty of eliminating catalyst residues. Consequently, researchers are directing their attention towards the utilization of enzyme catalysts [45]. The utilization of enzymatic catalysts will enhance biocompatibility, facilitate product purification, and provide enantioselectivity, regioselectivity, diastereoselectivity, and chemoselectivity, which are not afforded by chemical catalysis [46].

### 2.1. Chemical Synthesis

The current industrial synthesis process employs chemical reactions supported by catalysts due to their adjustable parameters and high efficiency, with Table 1 summarizing the resulting sugar esters’ degree of substitution (DS)/hydrophilic–lipophilic balance (HLB), critical micellar concentration (CMC), yield, reaction condition, and purity. Medium-polar solvents, such as dimethyl sulfoxide (DMSO) and dimethylformamide (DMF), were initially considered for the sugar ester (SE) reaction due to their excellent solubility in the substrate and broad application range [47]. The majority of synthetic surfactants are derived from fundamental chemical reactions, including sulfonation, ethoxylation, esterification, and alkylation. Of these, two processes are predominantly used in industrial applications [48]. Most commercially available compounds are anionic (negatively charged) or neutral and are preferred due to their higher biodegradability and lower toxicity compared to cationic (positively charged) or amphoteric (both positively and negatively charged) forms [49]. The principal substrates employed in chemical synthesis reactions are derived from either renewable or petrochemical sources [50,51]. The advancement of petrochemical processing, particularly petroleum cracking, has facilitated the production of hydrophobic surfactant components through the polymerization of alkenes such as ethylene and propylene, resulting in hydrophobic chains with a range of lengths, from C9 to C18. Traditional esterification methods for sugar-based surfactants involve the use of chemical catalysts [46]. Conventional esterification techniques for sugar-based surfactants typically employ the use of chemical catalysts. The synthesis of SEs entails the acylation and esterification of carbohydrates, encompassing mono-, di-, oligo-, and polysaccharides, in addition to polyols [52,53,54,55]. Such reactions occur with fatty acids or active carboxylic acid esters through transesterification [55,56]. It is of paramount importance that both reactions are conducted in a non-aqueous environment to prevent the formation of a non-ester reaction product [55]. The reactivity of fatty acids in these processes varies with chain length, typically ranging from 12 to 22 carbon atoms. The most common fatty acids are the saturated types, including lauric (C12), myristic (C14), palmitic (C16), and stearic (C18), as well as the unsaturated types, such as oleic (C18, monounsaturated), linoleic (C18, polyunsaturated), behenic (C22), and erucic (C22) [53,55,57].

A variety of chemical techniques are employed to enhance the selectivity of sugar ester synthesis products [56]. Two principal techniques are employed in this process: direct esterification, which involves the use of fatty acid chlorides or anhydrides, and transesterification, which utilizes fatty acid esters such as methyl ester and vinyl ester. It has been observed that elevated temperatures and the use of basic catalysts in sugar ester synthesis can result in discoloration of the product, as well as polymerization, cyclization, and dehydration of the reaction mixture, which in turn reduces the efficiency of the reaction [53]. The melting point of sugar esters can range from 40 to 79 °C. Melting point is very important for predicting the thermal properties of sugar esters during storage or industrial processes. Recent reports have shown that the use of sucrose octaacetate, 1–2% sodium metal as a catalyst, and FAME from vegetable oils in a three-necked reaction flask before the application of heat leads to the formation of a single-phase melt achieved 20–30 min after the application of heat. High HSE yields have been obtained at temperatures as low as 105 °C and synthesis times as short as 2 h, using a vacuum of 0–5 mm Hg [54]. Modifications are also employed in industry to eliminate toxic solvents utilized during synthesis. One such report involves the reaction between molten sucrose (mp 185 °C) and fatty acid methyl esters in the presence of lithium, potassium, and sodium soaps as solubilizers and catalysts at temperatures ranging from 170 °C to 187 °C. The optimal combination for the synthesis of sucrose fatty acid esters was determined through a series of experiments. The formulation involved the amalgamation of lithium oleate with sodium or potassium oleate, constituting 25% of the total soap content, calculated based on the weight of the sugar. This combination yielded the most effective sucrose fatty acid ester [54,58]. Simple esters are frequently prepared through esterification under Fischer conditions, utilizing appropriate alcohol and acid as starting materials. However, sucrose esters tend to form a mixture of glucose and fructose at a pH of 2–3 and a temperature of 70–80 °C, rendering transesterification of sucrose and fatty acids necessary under industrial conditions [59]. This process utilizes catalysts such as potassium carbonate or potassium soap, with N,N-dimethylformamide (DMF) frequently employed despite its high toxicity. Although dimethyl sulfoxide (DMSO) was also tested, the optimal conditions yielded an ester with a purity of only 50%. The high boiling points of these solvents (153 °C and 189 °C, respectively) present disposal challenges, which may affect the quality of the resulting ester [60]. A method for synthesizing sucrose esters with a low degree of substitution (DS) was described. This involved the use of sucrose, palm oil methyl ester (POME), an alkaline catalyst (K_2_CO_3_), and another sucrose ester (with DS 1–3) as non-ionic surfactants [55]. Furthermore, the esterification of sugars by acylation with anhydrous organic acids or acyl chlorides, and their derivatives, has been documented. The degree and arrangement of ester groups in the sugar moiety are dependent upon the reactivity of the sugar hydroxyl group and the structural properties of the acylating agent [61]. It was also demonstrated that lactose can be esterified in a regioselective manner using free acids, involving dicyclohexylcarbodiimide coupling, functional group removal, and hydrogenolysis of benzyl groups [46].

**Table 1:** Summary of methods of chemical synthesis of sugar esters.

Sugar	Substrate	Solvent	Catalyst	Product	CMC	DS/HLB	Yield [%]	Reaction condition	Purity [%]	Ref.
Sucrose	Methyl palmitate	H_2_O (1:1 *v*/*v*)	KOH	Sucrose palmitate ester	0.8757 g/cm^3^	5.47	39	100 °C, 2 h	90	[55,62]
	Ethyl laurate	DMSO (2:1 *v*/*v*)	K_2_CO_3_	Sucrose laurate	0.067 mol/L	0.364–6	78	70 °C, 2 h	92	[63]
	Acetic anhydride, coconut oil	DMF	K_2_CO_3_	-	38.6 dyne/cm	-	98	120–140 °C, 2–6 h	-	[64]
	Ethyl palmitate	DMSO (2:1 *v*/*v*)	K_2_CO_3_	Sucrose palmitate ester	1.5 × 10^−5^ mol/L	10–16	73	70 °C, 2 h	92	[55,63,65]
	Acetonitrile	DMSO (60/40 *v*/*v*)	K_2_CO_3_	-	-	-	40	140 °C	68	[66]
	3-Laurylthiazolidine-2-thione	Pyridine	NaH	-	-	-	72	22 °C, 15 h	-	[55]
	Palmitic acid	Methyl alcohol	CH3Ona	Sucrose palmitate ester	0.181 × 10^−3^ mol/L	-	76	65 °C, 2 h	90	[67]
	Methyl laurate	DMSO	Diaion PA306S	Sucrose laurate	-	-	98	90 °C, 3 h	70	[68]
	Palmitic benzoic anhydrite	Cyclohexane	Resin Amerlyst 15	Sucrose palmitate ester	-	-	63	4355 °C, 12 h	-	[69]
	Methyl stearate	DMF	KOMe	Sucrose stearate	-	-	81.6	120 °C, 20 min	-	[70]
	Vinyl palmitate	Bmim [dca]/2-methylbutan-2-ol (2M2B)	[Bu_4_N][Ac]	Sucrose palmitate ester	-	-	42	60 °C, 5 days	-	[71]
	Methyl laurate	DMSO	K_2_CO_3_	Sucrose laurate	-	-	69.89–73.28	80 °C	-	[72]
Glucose	Vinyl laureate	2M2B	[Bu_4_N][Ac]	Glucose laurate	-	-	33	40 °C, 30 h	-	[72,73]

Ionic liquids (ILs) are increasingly employed in esterification and transesterification reactions due to their distinctive properties, which include low volatility and high chemical and thermal stability [55,74]. These attributes render ILs highly suitable for a broad spectrum of applications, particularly in chemical synthesis, where they function as both solvents and catalysts. A notable application of ILs involves tetraalkylammonium salts, such as [Bu4N] [Ac], [Et4N] [Ac], and [Me4N] [Ac], which have been demonstrated to function as efficient solvents and catalysts in regioselective acylation processes. These reactions have successfully produced compounds such as glucose laurate, thereby demonstrating the potential of ILs in synthesizing complex molecules with high selectivity. The capacity of ILs to act as both solvents and catalysts simultaneously streamlines the reaction process, potentially reducing the necessity for additional chemical agents and simplifying the purification steps [73]. Moreover, research has indicated that ILs can facilitate the synthesis of sugar esters with a low degree of substitution without the necessity for enzyme catalysts. This is of particular benefit as it opens new avenues for chemical synthesis that are less reliant on biological catalysts, which can be costly and sensitive to reaction conditions. The dual role of ILs in such reactions highlights their multifaceted utility and positions them as valuable tools in the development of more efficient and sustainable chemical processes. Nevertheless, the recovery of products obtained using ILs presents a considerable challenge, despite the promising applications of these compounds. The high boiling points and stability of ILs complicate the separation and purification processes, often necessitating the use of sophisticated and potentially expensive techniques to retrieve the desired products in an efficient manner. It is of paramount importance to address these recovery issues if ILs are to be used in industrial applications on a practical and economical basis. The development of effective recovery and recycling methods for ILs will enhance their viability, thereby rendering them more attractive for widespread industrial use. In conclusion, the employment of ionic liquids in esterification and transesterification reactions offers substantial advantages due to their unique properties and multifunctional roles. While their application has led to significant advancements in chemical synthesis, ongoing research and development are necessary to overcome the challenges associated with product recovery and to fully achieve the potential of ILs in industrial processes [55,75,76,77,78].

### 2.2. Enzymatic Methods

Enzymatic glycosylation and transesterification (biocatalysis) have found widespread application in both laboratory and pilot-scale settings, particularly for the selective synthesis of sugar–fatty acid conjugates. These biocatalytic methods offer advantages such as mild reaction conditions, higher selectivity, and reduced environmental impact compared to conventional chemical synthesis. It employs lipases (e.g., *Candida antarctica* lipase B) or glycosyltransferases for regioselective acylation. While it demonstrates considerable promise for mild conditions and selectivity, the cost of enzymes and substrate purification remains a significant limiting factor for full-scale deployment [79].

It is increasingly recognized that microbial biosurfactants are sustainable, safe, and effective alternatives to synthetic or bio-based surfactants in cosmetic, personal care, and pharmaceutical applications [3]. Notwithstanding the prevailing tendency for elevated production costs, those derived from agro-industrial waste, including corn steep liquor, have been shown to be cost-competitive and environmentally beneficial [80]. This microbial biosurfactant is produced by a *Bacillus* strain capable of generating both extracellular and cell-bound surfactants, rendering it suitable for formulations such as hair care, skincare, sunscreens, and Pickering emulsions [81,82,83]. Nevertheless, challenges persist in the management of their biodegradation within formulations, with the objective of maintaining functionality whilst ensuring regulatory compliance through in vitro testing methods [84]. Furthermore, a significant proportion of contemporary cosmetic formulations comprise high concentrations of surfactants (up to 50%), which have the potential to induce skin irritation and give rise to environmental concerns [85]. It is evident that microbial biosurfactants are in alignment with the prevailing global trends towards green chemistry and natural ingredients. Their incorporation into commercial products has the potential to substantially mitigate the adverse health effects and environmental impact associated with conventional surfactants [3].

#### 2.2.1. Enzymatic Catalytic System

**Figure 3:**
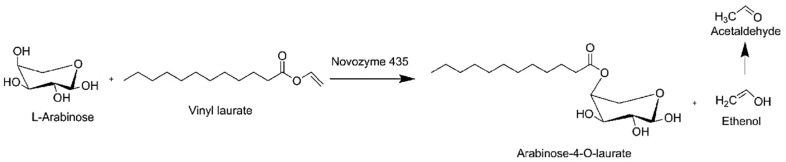
Example of lipase-catalyzed reaction for glycolipids (e.g., L-arabionose and vinyl laurate).

Enzymatic catalysis can be employed in the synthesis of sugar esters of both non-microbial and microbial origin. The utilization of enzymes provides a more straightforward purification process and the potential for reuse of the catalyst. The esterification of biomolecules is commonly carried out using enzymes such as lipases, esterases, and proteases [86,87,88]. Sugar fatty acid esters (SFAEs) are currently synthesized using enzyme catalysis as a greener alternative, given their end use as non-ionic and biodegradable emulsifiers and surfactants. Table 2 presents a synopsis of recent developments in the enzymatic esterification of sugars in the presence of ionic liquids. Ionic liquids (ILs) may be employed as substitutes for organic solvents in the synthesis of SFAEs [56,76]. The non-toxic and non-flammable nature of supercritical carbon dioxide (SC-CO_2_) has recently attracted the interest of researchers involved in the enzymatic synthesis of SFAEs. Furthermore, the insolubility of enzymes in SC-CO_2_ facilitates their separation [75,89]. Enzymatic catalysis represents a promising alternative to chemical synthesis, offering mild conditions and regioselectivity of the reaction [90]. Nevertheless, the utilization of solvents such as DMSO or DMF has been observed to result in low yields during the coupling reaction [91]. One potential solution is the use of a strongly polar alcohol doping solvent, such as 2-butanol, which has been demonstrated to result in the efficient production of succinic acid-6-acetate (57.42% yield) [55]. An alternative approach is the use of enzymes, such as Novozym 435, as a catalyst in conjunction with acyl donors in the form of vinyl acid esters, including vinyl hexanoate, vinyl octanoate, vinyl decanoate, vinyl laurate, vinyl myristate, vinyl palmitate, and vinyl stearate (Figure 3). This approach has been shown to enable the synthesis of seven distinct 6-O-acylglucose esters [90]. In summary, the utilization of enzymatic catalysis in the synthesis of sugar esters offers considerable advantages in terms of environmental sustainability and reaction specificity. The utilization of ionic liquids and supercritical carbon dioxide further enhances the potential for green chemistry in these processes. However, challenges such as solvent compatibility and product yields remain areas of active research and development.

Glycolipids, specifically N-acetyl-glucosamine fatty acid esters, have been synthesized through enzymatic catalysis, thereby demonstrating the potential of lipase-catalyzed reactions in producing these compounds. As previously reported, a lipase-catalyzed transesterification of methyl hexanoate with N-acetyl-glucosamine (GlcNAc) resulted in the formation of 2-(acetylamino)-2-deoxy-6-O-hexanoate-D-glucose. Furthermore, the use of N-butyryl-glucosamine (GlcNBu) in a similar synthesis led to the formation of 2-(butyryl amino)-2-deoxy-6-O-hexanoate-D-glucose [107]. The efficacy of enzymatic catalysis was further corroborated by a study that investigated the influence of fatty acid chain length (ranging from C4 to C12) on the esterification process catalyzed by *Candida antarctica* (Novozyme 435, EC 3.1.1.3). The reaction was conducted in a solvent mixture of tert-butanol and pyridine (9:11 *v*/*v*), resulting in the successful synthesis of maltose 6-O-acyl esters in an anomeric molar ratio of 1.0:1.1. These findings highlight the importance of lipases and proteases in the regioselective acylation of mono- and disaccharides [102]. Furthermore, the critical micelle concentration (CMC) and the efficiency in reducing the surface tension of water of nine different sugar monoesters derived from three disaccharides with varying carbon chain lengths (C8–C12) were examined. These esters were synthesized using immobilized lipase (Lipozyme TLIM). The study found that the CMC increased as the carbon chain length decreased, while the caprate monoesters demonstrated a lower surface tension [55].

#### 2.2.2. Solvent-Free System

To achieve a sustainable green technology approach, a number of strategies have been adopted, with a particular focus on the utilization of solvent-free systems in the synthesis of sugar esters [55,108]. These systems present numerous advantages, as the reaction environment consists solely of reagents, enhancing the volumetric productivity of the process and circumventing the generation of complex and hazardous waste products. The utilization of mobilized lipases in esterification reactions within solvent-free systems (SFS) has been demonstrated to be an effective approach, as evidenced by the literature [92,109]. The immobilization of the enzyme permits its recovery and reuse, which is associated with the potential for improved stability, activity, selectivity, or specificity of the enzyme [110]. In light of the toxicity of solvents employed in chemical synthesis and the complex nature of the final processing stages, a solvent-free system emerges as a preferable alternative, particularly in terms of the environmental impact. For example, Xie et al. presented a practical protocol for obtaining high-purity sucrose monostearate ester under solvent-free conditions, resulting in the synthesis of sucrose monostearate containing 74.6% of the product mixture, which is comparable to the best commercially available preparations [111]. Furthermore, solvent-free systems can extend the range of operational conditions, minimize the impact of chemical inhibition or inactivation, and facilitate purification processes. In the synthesis conducted using a solvent-free system, two parameters are of particular significance: the molar ratio and the catalyst loading. The reaction is thermodynamically stabilized, and thus the catalyst concentration determines the degree of conversion. The assessment of the molar ratio in SFS is of particular importance, given that the attainment of high conversions in the synthesis of the second substrate frequently necessitates the utilization of an excess reagent. The nature and quantity of the excess reagent in the system determines the critical physicochemical properties of the reaction environment at various stages of the reaction [112]. The reaction environment in this system is subject to a dynamic change in state. In the initial stages of the reaction, the components serve the function of reaction substrates. As the reaction progresses, the substrate is constituted by the remaining substrates, the ester product, and the by-product, namely water, unless it is removed from the system. The final reaction medium is typically characterized by higher hydrophobicity than that of the initial system. Moreover, the elimination of water from the system enables a transition in the thermodynamic profile of the process toward synthesis [108,113]. Nevertheless, the utilization of such systems is not without constraints. Notwithstanding the removal of water from the system, a residual quantity of water remains within the catalyst molecule, particularly when the enzyme is highly active. This can result in the formation of an aqueous phase within the enzyme, which subsequently reduces its reactivity. This phenomenon can be mitigated by the utilization of highly hydrophobic substrates or ultrasound [112,114]. Another challenge associated with this type of system is the degradation of chains and excessive losses during subsequent processing. Furthermore, following a change in solvents, the frequency of substrate contact is reduced due to the differing polarities of sugars and fatty acids [72]. Recent studies have investigated novel approaches to sugar acetylation, highlighting diverse catalysts and conditions for enhancing reaction efficiency and selectivity. Dysprosium (III) trifluoromethanesulfonate, Dy(OTf)3, has emerged as an effective catalyst for per-O-acetylation of unprotected sugars under solvent-free conditions, using near-stoichiometric amounts of acetic anhydride [115]. This method offers simplicity and efficiency in the protection of sugars for use in a variety of downstream applications. Giri et al. demonstrated the exceptional stability and utility of diazepinium perchlorate, an organic salt, in the acetylation of free sugars [116]. This neutral catalyst provides a robust alternative for selective acetylation reactions, ensuring high yields and minimal side reactions. Traboni et al. investigated the use of microwave-assisted silica sulfuric acid (SSA) as a catalyst for acetylation, specifically converting sialic acid methyl ester into per acetylated intermediates. The use of SSA under microwave irradiation has been demonstrated to enhance reaction kinetics and selectivity, illustrating its potential in efficient sugar modification [117]. The study by Ogawa et al. focused on the enzymatic synthesis of trehalose esters, with a particular focus on TRE (triacylglycerol esters), using Novozyme 435 in a solvent-free setup. Comparisons were made with glucose ester to highlight key reaction parameters. TRE was converted to trehalose mono ester (TME) and trehalose diester (TDE) through esterification with lauric acid (La) and transesterification with ethyl laurate (LaEt), respectively. Despite the presence of different by-products and boiling points (e.g., water vs. ethanol), both the TRE-La and TRE-LaEt systems require a minimum reaction temperature of approximately 90 °C. In contrast, the GLU-LaEt system operates effectively at 50 °C without the need for by-product removal [108]. To conclude, the utilization of solvent-free systems in the synthesis of sugar esters represents a notable advance towards the development of sustainable green technology. These systems offer several advantages, including enhanced process efficiency and a reduced environmental impact. However, there are still challenges to be overcome in optimizing reaction conditions and addressing issues related to enzyme activity. Further research and development in this area has the potential to result in more sustainable and efficient industrial processes.

#### 2.2.3. Synthesis in the Presence of DES

Deep eutectic solvents (DESs) represent a novel category of green solvents, formed by the mixing of two or more compounds, which results in a substance with a melting point lower than that of the individual components. When the compounds used to create DESs are primary metabolites, such as amino acids, organic acids, sugars, or choline derivatives, the resulting solvents are referred to as natural deep eutectic solvents (NADES). DESs and NADES have a range of applications, including in catalysis, organic synthesis, biotechnology, bioengineering, and electrochemistry [118,119]. The preparation of DESs typically involves the combination of a quaternary ammonium salt, such as choline chloride, with a hydrogen bond donor, including an amine, alcohol, amide, or carboxylic acid. A distinction can be made between DESs and ionic liquids (ILs) regarding the raw materials and the chemical processes employed in their preparation. The production of DESs is a relatively straightforward process, and they are derived from inexpensive and readily available compounds. Furthermore, they are considered to be more biodegradable, biocompatible, and sustainable than ILs, which makes them a valuable alternative [56]. The physicochemical properties of these molecules can be modified by the addition of different combinations of molecules, which allows them to be used in a wide range of applications. Consequently, they represent a versatile and intriguing medium for the study of biochemistry. DESs can be employed in the sustainable production of glycolipids, incorporating renewable resources such as lignocellulose. Lignocellulose, the most abundant renewable resource in the world, is subjected to a pretreatment process to produce sugar monomers, which serve as the starting materials for the enzymatic synthesis of surfactants. Following cleaning and drying, the glucose- and xylose-rich fractions are employed in the synthesis of DES, which is subsequently used to produce glycolipids. The utilization of DES derived from these sugars effectively addresses the issue of their low solubility in other anhydrous solvents, thereby ensuring the ready availability of sugars for reactions [120]. A multitude of review publications have delineated the prevailing trends and evolution of DESs, with a particular focus on their synthesis, properties, characteristics, and compositions [121]. Other publications have emphasized their significant potential in the field of nanotechnology, where they can be employed for the production of nanoscale and functional materials, the synthesis of biodiesel, the application of biocatalysis, the extraction and separation of compounds, and the study of electrochemistry [122]. DESs have been employed in a multitude of organic reactions, including the synthesis of polymers and related materials [123], lipase-catalyzed reactions, metal-catalyzed and metal-mediated organic reactions, biotransformation, organocatalysis, and multistep combinations of organ catalysts and enzymes [121]. A promising example of the innovative use of DES reaction media is the lipase-catalyzed synthesis of glycolipids, which can serve as detergents and biosurfactants. A two-in-one reaction system, comprising a DES composed of choline chloride and various sugars, has been successfully developed. In this enzymatic reaction, the sugars served the dual function of both the DES component and substrate. The utilization of ChCl DESs offers a distinct advantage in that it eliminates the necessity for organic solvents, while simultaneously resolving the issue of sugar solubility in non-aqueous solvents. A range of sugars in conjunction with different fatty acids have been investigated as prospective substrates for the enzymatic synthesis of bespoke glycolipids. Subsequent experiments demonstrated that DES systems could also be established with natural carbohydrate fractions derived from beechwood and with honey and agave syrup. This enabled the environmentally friendly enzymatic synthesis of bespoke glycolipids using only natural starting materials [124]. In a recent study, Girish et al. demonstrated the synthesis of polyethylene glycol stearate, a compound utilized in cosmetics and pharmaceuticals, via the use of DES and CALBex 10000. The highest conversion rate, 86.98%, was achieved within six hours. The yield of glucose monoester achieved was below 15%, which is likely due to the solvent’s characteristics. The use of DES as both a substrate and a solvent in the synthesis of glucose to 6-O-hexanoate with a DS of 1 was investigated. This entailed the mixing of ammonium salts and hydrogen donors in varying proportions at 100 °C, to elucidate the enzymatic synthesis of glucose hexanoate in a range of DESs [125]. The yield of glucose monoester achieved was below 15%, which is likely due to the solvent’s characteristics. The use of DES as both a substrate and a solvent in the synthesis of glucose to 6-O-hexanoate with a DS of 1 was investigated. This involved the mixing of ammonium salts and hydrogen donors in different proportions at 100 °C, with the aim of understanding the enzymatic synthesis of glucose hexanoate in various DESs [55]. In a study conducted by Hollenbach et al., the utilization of hydrophobic (-)-menthol acid DES was observed to markedly enhance the yield of glucose monodecanoate in comparison to the previously employed hydrophilic DES. Following 24 h, the hydrophilic DES (choline chloride) yielded 0.15 μmol/g DES (0.03%), whereas the hydrophobic DES demonstrated superior outcomes, resulting in higher yields. The (-)-menthol acid DES yielded 3.55 μmol/g DES (0.71%) with 0.5 M glucose and 18.73 μmol/g DES (3.75%) with 1.5 M glucose. These findings indicate that the polarity of the solvent is a crucial factor influencing glycolipid productivity [126].

Although microbial fermentation is a well-established method for producing glycolipid biosurfactants such as rhamnolipids and sophorolipids [56], the present review focuses on the chemo-enzymatic synthesis of sugar esters due to its advantages in structural control, product purity, and industrial scalability under defined conditions [120]. The production of microbial organisms is typically a complex process that necessitates the use of sophisticated media, protracted fermentation times, and a series of arduous downstream purification steps. Achieving standardization in these processes poses a considerable challenge for industrial surfactant applications [127]. In contrast, chemo-enzymatic approaches permit regioselective synthesis, reduced reaction times, and the ability to tailor the physicochemical properties of the final product by controlling the degree of substitution and fatty acid chain length [107].

### 2.3. Analytical Approaches for Structural Characterization and Quantification

In addition to the continuous progress in chemical and enzymatic synthesis of sugar esters, proper characterization and quantification of the resulting compounds remain crucial. Accurate analytical methods are essential not only for confirming the success of synthesis but also for determining the structural features, purity, degree of esterification, and potential application profiles of the products. Over the years, various analytical techniques have been developed and refined to meet these demands, allowing for a deeper understanding of sugar ester structures and functionalities.

**Figure 4:**
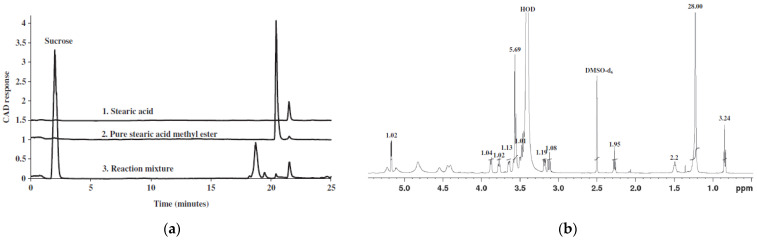
Characterization of stearic acid sucrose ester: (**a**) HPLC chromatograms of reactants and product [70]; (**b**) ^1^H NMR spectrum of the synthesized compound [70].

Early studies on sugar esters of fatty acids focused on their purification, structural elucidation of sugar moieties and esterified fatty acids, determination of acylation sites, the number of acyl groups, and quantification in crude plant extracts. Initial analytical efforts relied on techniques such as thin-layer chromatography (TLC), paper chromatography, elemental analysis, infrared (IR) spectroscopy, and chemical derivatization reactions [45]. The introduction of advanced analytical instrumentation has significantly enhanced the accuracy and resolution of sugar ester characterization. Currently, nuclear magnetic resonance (NMR) [70], electrospray ionization–mass spectrometry (ESI-MS) [44], and high-performance liquid chromatography (HPLC) [70] are commonly employed for structural analysis and quantification (Figure 4). In addition, enzymatic, colorimetric, and diverse chromatographic separation strategies have been implemented for metabolic profiling and functional studies.

In summary, the transition from classical methods to high-throughput, instrument-based techniques has profoundly improved our ability to characterize sugar esters of fatty acids. This analytical advancement provides a robust foundation for exploring their biological roles and chemical diversity.

## 3. Biological Activities

**Figure 5:**
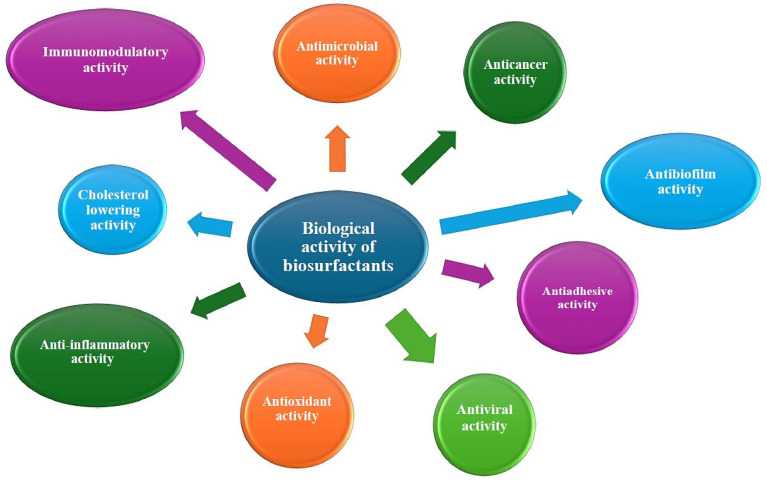
Biological activity of biosurfactants.

Biosurfactants represent a distinctive class of amphiphilic molecules produced by microorganisms. These molecules are capable of interacting with the lipid components of microorganisms, thereby modifying their physicochemical properties [128]. A substantial body of research has demonstrated that a considerable number of biosurfactants display a range of biological activities (Figure 5), including antimicrobial, antifungal, and anticancer effects [128,129,130].

Consequently, they offer considerable promise for use in a variety of applications within the medical, pharmaceutical, and agricultural sectors [131]. Glycolipids have demonstrated antibacterial activity against both gram-negative and gram-positive microorganisms, including *Staphylococcus aureus*, *Escherichia coli*, *Klebsiella pneumoniae*, and *Bacillus subtilis*, by damaging their cytoplasmic membranes [40]. In a recent study, Kyriakides et al. investigated the potential of D-mannose as a preventative measure for recurrent urinary tract infections (UTIs), suggesting that it may serve as an effective adjunct or alternative therapy [132]. The inhibitory activity of novel chloral-derived glucosamines was evaluated using agarose diffusion assays. The results demonstrated that 6-amino-6-deoxy-1,2-O-(S)-trichloroethylidene-α-D-glucofuranose exhibited moderate antimicrobial activity [133]. Ankulkar et al. demonstrated that semi-purified sophorolipids exhibited varying degrees of antibacterial activity against pathogenic *E. coli*, *Listeria monocytogenes*, and *S. aureus*, with minimum inhibitory concentrations (MICs) of 1000, 500, and 250 μg/mL, respectively [134]. Similarly, Fontoura et al. observed that gram-positive bacteria (*Enterococcus faecium*, *Staphylococcus aureus*, and *Streptococcus mutans*) exhibited greater sensitivity to sophorolipids than gram-negative bacteria (*Proteus mirabilis*, *E. coli*, *Salmonella enterica* subsp. *enterica*), with effective doses of 500 and 2000 μg/mL, respectively [135]. Glycolipids, a prominent type of biosurfactant, are renowned for their diverse bioactivities and substantial potential for practical applications, especially due to their environmental friendliness compared to chemical surfactants. An understanding of the fundamental antimicrobial mechanisms of glycolipids, which target microorganisms in both planktonic and biofilm states, provides a theoretical basis for the development of novel, safe antimicrobial agents for use in the food, cosmetics, and medical industries. Table 3 provides examples of glycolipids’ antimicrobial, antifungal, and anticancer applications.

Rhamnolipids have been demonstrated to inhibit the growth of significant pathogens, including *S. aureus* and *Staphylococcus epidermidis*. These biosurfactants impede the proliferation of planktonic cells at minimum inhibitory concentrations (MICs) of 0.06 and 0.12 mg/mL, respectively. The induction of bacterial cell death is attributed to cell lysis and the concomitant leakage of cellular components. The validity of this mechanism has been substantiated by the utilization of transmission electron microscopy (TEM) and scanning electron microscopy (SEM) imaging techniques. The incorporation of amphiphilic compounds into cell membranes has been demonstrated to result in alterations to the membrane’s structure, leading to an increase in membrane permeability and a concomitant reduction in membrane hydrophobicity and charge [148,149]. They can also disperse pre-formed biofilms by up to 93% [40]. The antimicrobial activity of sophorolipids has also been well documented. In a study conducted by Diaz de Rienzo et al., the antimicrobial properties and biofilm-disrupting capabilities of sophorolipids produced by *Candida bombicola* ATCC 22214 were investigated in both gram-negative and gram-positive bacteria. The findings revealed that at low concentrations (50 g/L), the sophorolipids exhibited fungicidal effects and effectively disrupted biofilms [150]. An innovative approach to preventing biofilm formation involves the pre-coating of medical devices with purified sophorolipids. This method has been demonstrated to inhibit the formation of biofilms by *Staphylococcus* spp. by reducing microbial cell attachment by 75% after a two-hour exposure period [151]. Adu et al. recently demonstrated that highly purified glycolipids exert differential effects on SK-MEL-28 cell lines, contingent on their chemical structure. The findings revealed that specific congeners, such as lactonic sophorolipids (SL) and mono-rhamnolipids (RL), were capable of inducing cell death and inhibiting the migration of melanoma cells while exerting a minimal impact on healthy skin cells [152]. In a recent study, Yang et al. demonstrated that a group of newly obtained polyglycolipids from *P. arsenia* rhizomes and glycolipids from *Pontenilla* can act as anti-inflammatory and hepatoprotective compounds by inhibiting 15-LOX. In addition, these compounds have been shown to possess protective effects against ROX. In addition, their utilization in in vitro studies has evidenced the capacity to impede pro-inflammatory cytokine secretion [153]. Sugar-based (thio)alkylglycosides exhibit antiproliferative properties against a human leukemia cell line. They also significantly influenced cytokine induction by RAW 264.7 macrophages. They also induced the induction of proinflammatory cytokines, particularly IL-1α and TNFα. Furthermore, they inhibited the proliferation and biofilm formation of clinical *C. albicans* strains at a concentration of 100 μg/mL [154]. Furthermore, the study found that glycolipids produced by *M. guilliermondii* exhibited anti-biofilm activity against *C. albicans* cells at a concentration of 4 mg/mL, with a resultant inhibition of biofilm formation of 80% [155]. A nanocomposite composed of a rhamnolipid and a C60 molecule demonstrated the ability to scavenge free radicals by 80% for NO and 40% for OH at a concentration of 0.4 mg/mL. In addition, in vivo studies have demonstrated the capacity of these molecules to immunoregulate, resulting in a substantial reduction in the levels of proinflammatory cytokines, hemoproteins, and oxidoreductases present in ASUC. Moreover, the compounds are not toxic due to CCK-8 assay [156]. Rhamnolipids have also been shown to possess antineoplastic properties, with the capacity to inhibit the proliferation of cervical cancer cell line (Hela) and L20B (poliovirus receptor CD155). The median inhibitory concentration (IC50) for Hela cells was 62.5 μg/mL, while for L20B cells it was 750 μg/mL [157].

## 4. Conclusions

The utilization of surfactants derived from sugars and fatty acids has increased significantly in recent times due to their unique properties and the wide-ranging applications they offer in various industries, including nutrition, cosmetics, and pharmaceuticals. Despite the advantages these compounds offer, the conventional chemical synthesis of such materials presents a number of environmental challenges, including the toxicity of solvents and the complexity of purification processes. This review emphasizes the increasing interest in the development of environmentally friendly synthesis methods, including the use of enzymatic catalysis and the employment of green solvents such as deep eutectic solvents (DES). The use of enzymes in synthesis offers a number of advantages, including greater specificity, simplified purification processes and a reduced environmental impact. Nevertheless, challenges such as low yields and solvent compatibility persist. DESs, constituted from biodegradable and biocompatible components, present a promising alternative to traditional solvents. These solvents not only reduce environmental toxicity but also address the solubility issues of sugars in non-aqueous environments, rendering them suitable for the synthesis of glycolipids. The utilization of DESs has demonstrated the potential to enhance the efficiency and sustainability of the synthesis process. However, their high viscosity can present practical challenges. Moreover, the biological activities of sugar-based surfactants, including their antimicrobial, antifungal, and anticancer properties, underscore their potential for use in a range of medical, pharmaceutical, and agricultural applications. Glycolipids, for instance, have exhibited notable antibacterial and anti-biofilm properties, rendering them applicable in diverse industrial contexts. In conclusion, the advancement of sustainable industrial practices hinges on the development and optimization of greener synthesis methods for sugar-based surfactants. It is imperative that continued research and innovation in enzymatic catalysis and the application of DESs be pursued in order to overcome existing challenges and fully achieve the potential of these environmentally friendly surfactants.

## Figures and Tables

**Table 2:** Recent reports of enzymatic esterification of sugar esters.

Sugar	Substrate	Enzyme	Solvent	Product	Yield[%]	Purity [%]	Ref.
Trehalose	Palmitic, lauric, and caprylic acids	*Candida antarctica* lipase B (CALB 10000)	Acetone	Trehalose fatty acid esters (THFAE)	34.55	-	[45]
Glucose and xylose	Vinyl laurate	Novozyme 435	t-amyl alcohol	Glucose laurate and xylose laurate	87	96	[92]
Cy3glc (from blueberry)	Fatty acid C-10	CALB	t-amyl alcohol	Fatty acid esters of cyanidin-3-glucoside	25–47	95	[93,94]
Xylose	Vinyl laurate	Novozyme 435	t-amyl alcohol	Xylobiose, xylotriose, xylotetraose laurate	86	-	[95]
Glucose	Fatty acid	CALB and *Candida rugosa* lipase (CRL)	DMSO: t-amyl alcohol (80:20 *v*/*v*)	Glucose fatty acid esters	80.3	-	[96]
Glucose, xylose	Lauric acid, methyl laurate	Novozyme 435	t-amyl alcohol	D-glucose laurate and D-xylose laurate	65	-	[97]
Maltoheptaose	Palmitic acid, myristic acid, lauric acid, capric acid, and *p*-nitrophenyl laurate	CALB	10% DMSO	Maltoheptaose palmitate	64.60	-	[98]
Sucrose	Vinyl caprate	Lipase AY	DMSO, Crown ether	Sucrose caprate	68	22	[72]
D-maltose	Vinyl laurate	Novozyme 435	THF/pyridine (7:3 *v*/*v*)	6ʹ-O-Lauroylmaltose	80	-	[99]
Fructose	Lauric acid	CALB	Tert-amyl alcohol	Fructose laurate	70	-	[100]
Glucose	Vinyl laurate	Novozyme 435	[Bmim][Tf2N]/[Bmim][TfO]	Glucose laurate	64	-	[101]
Glucose	Vinyl laurate	CALB	[Bmim][BF4]/tert-BuOH (60:40%)	Glucose laurate	96.4	-	[72]
Galactose	Oleic acid	Lipozyme RMIM	DMSO: [Bmim][BF4] (1:20 *v*/*v*)	Galactose oleate ester	77	87	[72]
Glucose	Lauric acid	NZ435	n-hexane SCCO_2_	Glucose laurate	-	-	[102]
L-rhamnose	Vinyl laurate	*Pseudomonas stutzeri* lipase	Tetrahydrofuran	4-O-lauroylrham	58	-	[103]
Sucrose	Erucic acid	Lipozyme TLIM	T-butanol, DMSO (4:1)	Sucrose erucate	52.47	40	[72]
Sucrose	Stearic acid	Novozyme 435	Isooctane	Sucrose stearate	97.1	95	[104]
Sucrose	Vinyl laurate	Lipozyme TLIM	2M2B: DMSO (4:1)	Sucrose laurate	-	-	[72]
Sucrose	Vinyl laurate	Lipozyme TLIM	[3CIM(EO)] [NTf2]/[2M2B]	Sucrose laurate	70–90	-	[105]
Sucrose	Oleic acid	CALB	Solvent- free	Sucrose oleate	81–83	70	[72]
Xylose	Oleic acid	Novozyme 435	Methyl ethyl ketone	Xylose oleate	80	16.3	[106]

**Table 3:** Biological activity of sugar-based surfactants.

Surfactant/Biosurfactant	Biological Activity	Ref.
Mannosyl erythritol lipids	Anti-melanogenic	[136]
Mannosyl erythritol lipids	Antibacterial	[137]
Sophorolipids	Anti-biofilm	[137]
Mono- and di-rhamnolipids	Antimicrobial	[138]
Mono- and di-rhamnolipids	Cytotoxic effect on human breast cancer	[130]
Mono- and di-rhamnolipids	Cytotoxic effect on HL-60, SKW-3, JMSU-1, B-173	[139]
Rhamnolipids	Antifungal activity against fungi such as *Alternaria alternata*, *Mucor circinelloides*, and *Verticillium dahlia*	[139]
Rhamnolipids	Antimicrobial, anti-biofilm against *Bacillus subtilis*	[140]
Sophorolipids	Antibacterial, antimicrobial, and anti-adhesive towards *Escherichia coli*, *Bacillus subtilis*, *Staphylococcus aureus*, and *Campylobacter jejuni*	[131,141]
Sophorolipids	Anti-phytopathogens, antifungal applications	[142,143]
Rhamnolipid	Anti-cancer and autophagy inhibitors	[144]
Rhamnolipid	Anti-bacterial effects on *Staphylococcus aureus*, *Klebsiella pneumonia*	[145]
Rhamnolipid	Anti-viral effects on herpesvirus, tobacco mosaic virus (crop viral infection), bovine coronavirus, SARS-CoV-2	[146,147]
Sophorolipids	Anti-viral effects on HIV, Epstein–Barr virus, and influenza virus	[147]

## Data Availability

No new data were created or analyzed in this study.
